# Cross Strain Protection against Cytomegalovirus Reduces DISC Vaccine Efficacy against CMV in the Guinea Pig Model

**DOI:** 10.3390/v14040760

**Published:** 2022-04-06

**Authors:** K. Yeon Choi, Nadia S. El-Hamdi, Alistair McGregor

**Affiliations:** Department Microbial Pathogenesis & Immunology, College of Medicine, Texas A&M University, Bryan, TX 77807, USA; yeonchoi@tamu.edu (K.Y.C.); nselhamdi@tamu.edu (N.S.E.-H.)

**Keywords:** guinea pig, cytomegalovirus, glycoproteins, neutralizing antibody, congenital CMV, pentamer complex, gB, epithelial cells, virus tropism, disabled infectious single cycle (DISC)

## Abstract

Congenital cytomegalovirus (CMV) is a leading cause of disease in newborns and a vaccine is a high priority. The guinea pig is the only small animal model for congenital CMV but requires guinea pig cytomegalovirus (GPCMV). Previously, a disabled infectious single cycle (DISC) vaccine strategy demonstrated complete protection against congenital GPCMV (22122 strain) and required neutralizing antibodies to various viral glycoprotein complexes. This included gB, essential for all cell types, and the pentamer complex (PC) for infection of non-fibroblast cells. All GPCMV research has utilized prototype strain 22122 limiting the translational impact, as numerous human CMV strains exist allowing re-infection and congenital CMV despite convalescent immunity. A novel GPCMV strain isolate (designated TAMYC) enabled vaccine cross strain protection studies. A GPCMV DISC (PC+) vaccine (22122 strain) induced a comprehensive immune response in animals, but vaccinated animals challenged with the TAMYC strain virus resulted in sustained viremia and the virus spread to target organs (liver, lung and spleen) with a significant viral load in the salivary glands. Protection was better than natural convalescent immunity, but the results fell short of previous DISC vaccine sterilizing immunity against the homologous 22122 virus challenge, despite a similarity in viral glycoprotein sequences between strains. The outcome suggests a limitation of the current DISC vaccine design against heterologous infection.

## 1. Introduction

Human cytomegalovirus (HCMV), a betaherpesvirus, is a leading cause of congenital infection resulting in serious symptomatic diseases including cognitive and vision impairment as well as hearing loss in newborns [1,2]. Sensorineural hearing loss (SNHL) is the most common disease associated with congenital CMV and can continue to develop after birth [3]. Globally, congenital CMV occurs in approximately 1–5% of live births and this includes areas with high CMV seropositivity [4]. Primary CMV infection during pregnancy carries the greatest risk [4,5], but congenital CMV can also occur in women convalescent for the virus, and in these cases congenital infection may result from infection by a new strain [4]. Consequently, congenital infection can occur in mothers both seropositive and seronegative prior to pregnancy [6]. Ideally, a vaccine against congenital CMV should provide protection at the level higher than convalescent immunity to enable protection against infection by new strains of the virus.

Although the correlates of protection against congenital HCMV are poorly defined, it is generally thought that neutralizing antibodies to viral glycoprotein complexes significantly contributes to protection, but immune protection can also be enhanced by response to T cell target antigens (e.g., pp65) in convalescent immunity [7]. The evaluation of intervention strategies against CMV in a preclinical animal model is complicated by the species-specific nature of HCMV, making direct study of infection in animal models untenable. Species-specific animal CMV crosses the placenta in both the rhesus macaque (rhesus cytomegalovirus virus, RhCMV) and guinea pig (guinea pig cytomegalovirus, GPCMV) [8,9]. The guinea pig is the only small animal model for congenital CMV and the focus of this paper. Importantly, congenitally infected newborn pups have similar disease symptoms as humans, e.g., SNHL [10]. Various vaccine and intervention strategies have been evaluated against CMV in this model but studies have focused on the original prototype GPCMV strain 22122 (ATCC VR682) isolated in the 1950s [11]. Although this virus causes congenital infection, it is the only strain used in vaccine protection studies and was passed on fibroblast cells (>100) during the initial isolation which potentially attenuated the virus in contrast to clinical strains present in animal colonies. We recently isolated a new strain of GPCMV (designated TAMYC) from the salivary gland of an infected animal. This novel strain enabled the realistic evaluation of cross strain protection provided by promising CMV vaccine candidates against the 22122 strain in this translational animal model [12].

HCMV encodes multiple glycoprotein complexes (gB, gH/gL/gO, gM/gN and the pentamer complex (PC) gH/gL/UL128/UL130/UL131) important for cellular infection. The virus has two pathways of cell entry: direct entry which is independent of the pentamer complex (PC); and endocytic entry which requires PC in addition to other viral glycoproteins. The viral gB glycoprotein is essential for HCMV entry into all cell types, an immunodominant neutralizing antibody target, and remains a significant focus in various vaccine approaches, either as a standalone antigen, or in conjunction with other target viral antigens [13]. Although the gB protein generates neutralizing antibodies, these are less effective against virus neutralization on non-fibroblast cells including epithelial, endothelial and placental trophoblast cells where the viral pentamer complex (PC) is necessary for virus cell entry and the PC is an effective neutralizing antibody target [14,15]. In clinical trials, a subunit gB vaccine attains at best about 50% efficacy despite vaccine enhancement from non-neutralizing antibodies [16,17]. Consequently, the PC is also currently being evaluated in various CMV vaccine strategies as an important antigen target.

GPCMV encodes functional viral glycoprotein complexes to HCMV (gB, gH/gL/gO, gM/gN), which are important for virus cell entry [18,19,20]. Unlike murine cytomegalovirus, GPCMV also encodes a gH-based PC (gH/gLGP129/GP131/GP133) which is essential for GPCMV infection of all non-fibroblast cell types including epithelial and endothelial cells via an endocytic entry pathway similar to clinical strains of HCMV [19,20,21]. The PC is necessary for GPCMV dissemination and infection of placental trophoblasts and amniotic sac cells as well as congenital CMV [19,22,23]. As with HCMV, the GPCMV viral glycoprotein complexes are important neutralizing antibody targets [18,24,25,26,27]. GPCMV gB [25,28] is essential for virus infection of all cell types [18,19] and has been the most extensively studied vaccine antigen against congenital CMV in guinea pigs. However, in congenital protection studies, the various gB vaccine studies attained approximately 50% efficacy in the guinea pig model [28,29,30,31]. In HCMV, the endocytic pathway for cell entry is only partially defined and various candidate receptors have been identified [32,33,34,35,36]. Fibroblast cells that express the viral cell receptor platelet derived growth factor receptor alpha (PDGFRA) enable HCMV and GPCMV cell entry by direct cell fusion independent of the PC, but require gH/gL/gO triplex and gB [37,38,39]. Despite the essential nature of gB for infection of all cell types for HCMV and GPCMV, neutralizing antibodies directed to the PC might constitute a better vaccine target [40,41]. This is especially the case since antibodies directed to the PC are more effective at virus neutralization on placental trophoblasts and amniotic sac cell lines [14,23,27,38,42].

In addition to neutralizing antibodies, convalescent HCMV patients produce T cell responses to additional viral antigens including pp65 tegument protein and IE1 non-structural protein which are also thought to contribute to convalescent immunity [7,43]. Studies in animal models suggest that homologs of these antigens can also contribute to CMV vaccine-based protection. Consequently, the most effective CMV vaccine strategy might be one that evokes an immune response to various antibody and T cell target antigens. This potentially requires a complicated series of antigens to be present in candidate CMV vaccines to ensure a high level of efficacy. GPCMV encodes homolog T cell target antigens to HCMV such as pp65 (GP83), and a cell-mediated response to GP83 has been demonstrated to provide partial protection against congenital CMV [24,44,45], but has a limited impact as a standalone vaccine candidate [45,46]. Although various vaccine strategies have been evaluated in the guinea pig against congenital CMV, the most effective approach to date has been the use of a replication incompetent live viral strain or disabled infectious single cycle (DISC) vaccine [24]. The GPCMV DISC vaccine incorporates various antibody and T cell target antigens mimicking natural infection, but does not produce progeny virus in the host because of a lethal capsid gene mutation, and requires a complementing cell line for growth [24,38]. Protection against wild type virus (22122) challenge both horizontally and vertically was significantly increased with high efficacy and sterilizing immunity when this DISC vaccine strategy incorporated the unique PC components (GP129, GP131 and GP133) [38] compared to a GPCMV DISC (PC^−^) vaccine that expressed only gH and gL but lacked the unique PC components [24]. A PC^+^ DISC vaccine for HCMV based on a targeted viral protein destabilizing strategy is currently being evaluated in clinical trials [47].

In this current study, we hypothesized that a newly isolated strain of GPCMV (TAMYC strain) [12] may better resemble clinical strains present in guinea pig colonies. Consequently, this novel strain might provide a more significant test for CMV vaccine efficacy by evaluating heterologous cross strain vaccine protection in this model compared to previous homologous studies with the 22122 strain. Importantly, the TAMYC strain virus was highly cell associated similar to HCMV clinical strains and exhibited preferential tropism to various non-fibroblast cell types compared to the 22122 strain GPCMV [12]. An earlier cross strain protection study with a recombinant AdgB (22122 strain) vaccine failed to provide high level protection against the TAMYC virus challenge despite 99% identity in the gB sequence between strains [27,48]. This indicated a potential requirement for an immune response to multiple viral antigens for cross strain protection to be effective. Consequently, we evaluated the ability of the PC^+^ 22122 strain based GPCMV DISC vaccine (designated DISCII) to cross protect against a challenge by the novel GPCMV strain (TAMYC) in vaccinated animals as this strategy had exhibited sterilizing immunity against the 22122 strain and induced a comprehensive immune response. Additionally, the ability of convalescent and hyperimmune animals (22122 strain) to protect against infection by the TAMYC strain GPCMV challenge was explored. Subsequently, antibody neutralization of both TAMYC and 22122 strains were separately evaluated with hyperimmune sera (TAMYC or 22122 strain infected animals) to ascertain differences between homologous and heterologous virus neutralization on fibroblast and epithelial cells. Overall, the results suggest a minimum threshold expectation for an effective vaccine strategy that exhibits cross strain protection against CMV in this model.

## 2. Materials and Methods

### 2.1. Virus, Cells, Synthetic Genes and Oligonucleotides

Wild type GPCMV (strain 22122, ATCC VR682 or new strain isolate, designated TAMYC) were propagated on guinea pig fibroblast lung cells (GPL; ATCC CCL 158) and renal epithelial (REPI) cell lines as previously described [19,22]. Both 22122 and TAMYC strain viruses were PC positive. Virus stocks for antibody neutralization assays were generated on renal epithelial cells. Virus titers were determined by GPCMV titration on renal epithelial and fibroblast cells [19]. Recombinant defective adenovirus (Ad5) vectors encoding GPCMV glycoproteins (gB, gH, gL, GP129, GP131 and GP133) were previously described [18,19,27]. Oligonucleotides were synthesized by Sigma-Genosys (The Woodlands, TX, USA).

### 2.2. Animal Studies

Guinea pig (Hartley) animal studies were performed under IACUC (Texas A&M University, College Station, TX, USA) permit 2017-0227. All study procedures were carried out in strict accordance with the recommendations in the “Guide for the Care and Use of Laboratory Animals of the National Institutes of Health”. Animals were observed daily by trained animal care staff, and animals that required care were referred to the attending veterinarian for immediate care or euthanasia. Terminal euthanasia was carried out by lethal CO_2_ overdose followed by cervical dislocation in accordance with IACUC protocol and NIH guidelines. Animals purchased from Charles River Laboratories were verified as seronegative for GPCMV by anti-GPCMV ELISA of sera collected by toenail clip bleed as previously described [18]. Animal studies were performed to evaluate: (a) immune response to GPCMV infection; (b) virus dissemination in seropositive and seronegative animals; (c) vaccine protection against GPCMV infection. Animals were made immune to GPCMV by single injection (SQ, 1 × 10^5^ pfu) or hyperimmune by 3 sequential injections of the same strain of GPCMV with each injection separated by 3–4-week intervals. Infected animals were bled by toenail clip and serum from individual animals evaluated for anti-GPCMV titer by ELISA to verify seroconversion. Anti-glycoprotein complex ELISA titers (gB, gH/gL, gM/gN, PC) were also evaluated at approximately 2 months post final virus injection/vaccination. Sera of animals within each group with similar anti-GPCMV ELISA titers were pooled for further study. Hyperimmune pooled anti-GPCMV sera was previously described [24]. The antibody immune response to specific glycoprotein complexes (gB, gH/gL, gM/gN and PC) was evaluated by ELISAs for pooled sera for each group following the previously described assays [38]. Neutralization assays were evaluated on fibroblast and renal epithelial cells as previously described [38] for pooled sera from each group or seronegative control sera. Animals were vaccinated with DISCII (strain 22122) GPCMV (SQ 1 × 10^3^ pfu) followed by two sequential DISCII vaccine booster shots as described for hyperimmune animals. DISCI (PC negative) vaccine sera was historical pooled sera as previously described [38].

### 2.3. GPCMV Glycoprotein ELISAs

Specific glycoprotein complex ELISAs were carried out as previously described using positive coating antigen derived from renal epithelial cell monolayers transduced with recombinant replication defective adenovirus (Ad) vectors expressing specific glycoprotein complexes, or control recombinant Ad vectors expressing GFP for negative coating antigen [18,19,24,27]. This was except for the case of gM/gN ELISA which utilized transient expression plasmid with synthetic codon optimized expression plasmids for transfection onto guinea pig cells [18]. Harvested cells were washed with PBS and cell pellets fixed prior to processing as coating antigen. Protein concentration was normalized by a Bradford assay. MaxiSorp ELISA plates (NUNC) were coated with 0.5 µg of either Ag+ or Ag- preparations diluted in carbonate coating buffer overnight at 4 °C, washed in 1X PBST then blocked with 2% nonfat dry milk. Test sera were diluted in blocking buffer from 1:80 to 1:20,480 in doubling dilutions, incubated for 2 h at 37 °C and then reacted with anti-Guinea Pig IgG peroxidase antibody (Sigma-Aldrich, St. Louis, MO, USA) diluted (1:2000) in blocking buffer for an additional 1 h at 37 °C before reacting with TMB membrane peroxidase substrate (KPL). Net OD (absorbance 450 nm) was attained by subtracting the OD of Ag- from the OD of Ag+. All ELISAs described in this report were carried out with the same batch of coating antigen. The described approach is based on similar strategies for glycoprotein complex expression for HCMV and RhCMV and ELISAs [49,50]. All ELISAs were run a minimum of three times in duplicates. ELISA reactivity was considered positive if the net OD was greater than, or equal to, 0.2, as determined by GPCMV negative serum.

### 2.4. GPCMV Neutralization Assays

GPCMV neutralization assays (NA_50_) were performed on GPL fibroblasts and renal epithelial (REPI) cells with PC^+^ GPCMV (22122 strain or TAMYC strain) virus stocks generated on renal epithelial cells [18,19] using pooled sera from a specific group of GPCMV convalescent or DISC vaccinated animals as previously described [24]. Serially diluted sera were incubated with approximately 1 × 10^5^ pfu PC^+^ GPCMV in media containing 1% rabbit complement (Equitech Bio, Kerrville, TX, USA) for 90 min at 37 °C before infecting REPI cells for 1 h. For neutralization on GPL cells, 1 × 10^3^ pfu PC^+^ GPCMV was used. Infected cells and supernatant were collected on day 4 then titrated on GPLs. The final neutralizing antibody titer was the inverse of the highest dilution, producing 50% or greater reduction in plaques compared to the virus only control. NA_50_ were performed from each sample three times concurrently with the same virus stocks between groups.

### 2.5. Real Time PCR

Blood and tissues (lung, liver, spleen, salivary gland) were collected from euthanized guinea pigs to determine the viral load as previously described [18,24]. Briefly, for tissue DNA extraction, FastPrep 24 (MP Biomedicals, Solon, OH, USA) was used to homogenize tissues as a 10% weight/volume homogenate in Lysing Matrix D (MP Biomedicals). To obtain DNA from whole blood, blood was collected into tubes containing ACD anticoagulant and 200 μL of blood was subsequently used per extraction. DNA was extracted using the QIAcube (Qiagen Inc., Germantown, MD, USA) according to the manufacturer’s liquid (blood) or tissue protocol instructions. Viral load was determined by real time PCR on LightCycler 480 (Roche Life Science, Indianapolis, IN, USA) using primers and a hydrolysis probe to amplify a product from the GPCMV GP44 gene. The PCR master mix contained LightCycler ProbesMaster (Roche Life Science, Indianapolis, IN, USA), 0.4 μM primers and 0.1 μM probe, and 0.4U uracil N-glycosylase (UNG) in 25 μL of the total reaction volume including 10 μL of DNA per reaction. Standard controls and no template controls (NTC) were run with each assay for quantification. LightCycler 480 amplification parameters were: UNG step for 10 min at 40 °C followed by activation at 95 °C for 10 min, then 45 cycles of denaturation at 95 °C for 15 s, annealing at 56 °C for 15 s, and elongation at 72 °C for 10 s. Data were collected by ‘single’ acquisition during the extension step. The standard curve was generated using the GPCMV GP44 plasmid [51] for quantification and assay sensitivity. The sensitivity of the assay was determined to be 5 copies/reaction. The viral load was expressed as genome copies/mL of blood or genome copies/mg tissue. Results calculated were a mean value of triplicate PCR runs per sample.

### 2.6. Statistical Analysis

All statistical analyses were conducted with GraphPad Prism (version 7) software. Replicate means were analyzed using Student’s *t* test (unpaired), with significance taken as a *p* value of <0.05 or as specified in the figure legends.

## 3. Results

### 3.1. Comparative Ability of GPCMV DISC Vaccine (PC^+^ or PC^−^) to Neutralize TAMYC Strain

Previously, we demonstrated that inclusion of the PC in a GPCMV DISC vaccine strategy improved virus neutralization as well as vaccine efficacy against prototype strain 22122, providing sterilizing immunity against dissemination and congenital infection [38]. The ability of a DISC vaccine strategy to provide cross strain GPCMV protection was unknown and a limitation of the current model. A new strain of GPCMV (TAMYC) was isolated from the salivary gland of an infected commercial colony animal. A comparison of encoded GPCMV glycoprotein indicated that apart from the gO glycoprotein there was relatively high similarity between the respective strain glycoproteins at the predicted amino acid level and percentage homology [12]: gB (99%); gN (92%); gO (75%); gH (84%); gM (99%); gL (98%); GP129 (88%); GP131 (89%); GP133 (91%). Consequently, cross strain protection against the TAMYC strain was potentially possible with the current PC^+^ GPCMV DISC (22122) vaccine strategy, DISCII [38].

**Figure 1:**
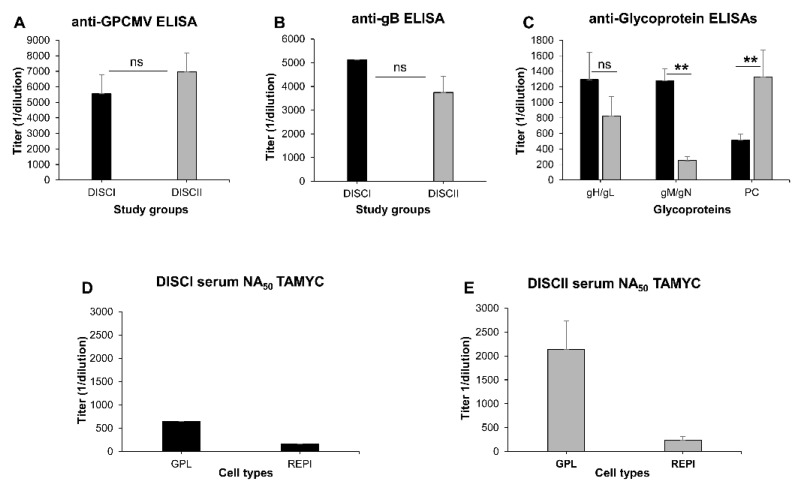
Comparative immune responses to DISC vaccine (DISCI vs. DISCII) and GPCMV (TAMYC strain) neutralization on fibroblast and epithelial cells. (**A**–**C**) Immune response of DISCII animal sera was compared to that of previous historical DISCI pooled sera from animals vaccinated with identical protocol [38]. (**A**) Anti-GPCMV ELISA titer; (**B**) anti-gB glycoprotein ELISA titer; (**C**) anti-glycoprotein complex ELISA titers (gH/gL, gM/gN, PC) from sera of DISCI (black) or DISCII (gray) vaccinated animals. Neutralizing antibody titers (NA_50_) against TAMYC strain virus on GPL (fibroblast) or REPI (epithelial) cells of pooled sera from either DISCI animals (**D**) or DISCII-vaccinated animals (**E**). Mean ELISA and neutralization values are a result of assay triplicates with each sample run a minimum of three independent times. Statistical analysis was determined by unpaired Student’s *t* test; ** *p* < 0.005; ns = non-significant.

A group of GPCMV seronegative female animals (*n* = 12) were vaccinated with a sequential three-dose vaccine regime of the DISCII vaccine (1 × 10^3^ pfu/shot, SQ). The first vaccine was given at day 0, followed by a booster at days 26 and 49-post original shot. Animals were bled at 24 and 48 days after original day 0 to determine anti-GPCMV antibody titer. On day 70, a final bleed was used to evaluate immune response to specific glycoprotein complexes (gB, gH/gL, gM/gN and PC) [18,24]. Historical pooled sera from 22122 strain DISCI (PC^−^) vaccinated animals [38] generated under the same vaccine regime to DISCII were compared with DISCII vaccine sera for immune responses to GPCMV glycoprotein complexes, and additionally evaluated for virus neutralization (TAMYC strain). Sera antibody immune response comparisons between the DISCI and DISCII vaccine strategies are shown in Figure 1: anti-GPCMV ELISA (Figure 1A); anti-glycoprotein complex ELISAs (Figure 1B,C).

DISCII vaccine results were similar to that previously observed in DISCII vaccinated animals [38] and included a specific response to PC. DISCII induced a higher anti-GPCMV ELISA titer compared to historical DISCI sera (5947 vs. 6950) but was not significant (Figure 1A). DISCI vaccine sera induced slightly higher anti-gB antibody titers compared to DISCII (5120 vs. 3750) but was not statistically significant (Figure 1B). However, DISCI vaccine sera induced approximately five-fold higher anti-gM/gN titer compared to DISCII, but anti-gH/gL titers were more similar between DISC vaccines (Figure 1C). Next, DISC vaccine sera were evaluated for their ability to neutralize the TAMYC strain GPCMV on both fibroblast (GPL) and epithelial (REPI) cells. Both DISCI and DISCII sera were more effective at neutralizing the virus on GPL cells (Figure 1D,E), with DISCII having a higher NA_50_ than DISCI (2133 vs. 640). This was despite the essential nature of gB, higher anti-gB titer in DISCI sera and 99% identity in gB sequence between TAMYC and 22122 strains. Both DISCI and DISCII sera had reduced neutralizing titers on epithelial cells with approximately four and nine-fold reductions, respectively, compared to on GPL cells (Figure 1D,E). Previous studies with 22122 neutralization and DISC vaccine sera indicated that inclusion of the PC improved virus neutralization on both fibroblast and non-fibroblast cells, and this would appear to be a similar outcome against the TAMYC virus (Figure 1D,E) [38]. Previously, depletion of antibodies to specific viral glycoprotein complexes from DISCII sera and 22122 hyperimmune GPCMV (PC^+^/PC^−^) sera [38] demonstrated that improved virus neutralization on non-fibroblast cells was attributed to anti-PC antibodies. The presence of anti-PC improved the DISCII virus NA_50_ titer against the TAMYC virus, but this was only two-fold greater than that of DISCI sera (Figure 1D,E) and suggests a more limited impact of anti-PC against the TAMYC strain. This might relate to similar levels of anti-gH/gL antibodies generated in both DISC vaccine strategies. Possibly gH/gL might be present on the virion surface of TAMYC in a greater level than PC compared to the 22122 strain, despite both virus stocks being generated on epithelial virus. The levels of specific viral glycoprotein complexes related to gH/gL have been reported to differ between HCMV strain types related to gO strain type, and this might impact on specific neutralizing antibodies titer directed to PC [52,53,54].

### 3.2. DISC Vaccine Cross Protection against GPCMV (TAMYC Strain) Virus Challenge

**Figure 2:**
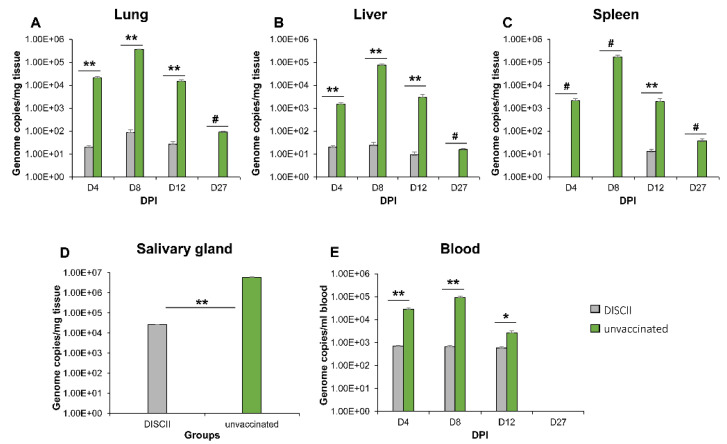
DISCII vaccine fails to prevent dissemination of heterologous GPCMV (TAMYC strain) to target organs in vaccinated animals. Seronegative animals (*n* = 12) were vaccinated with 3 sequential shots of DISCII vaccine. Animals were evaluated for immune response (Figure 1) and at 3 weeks post-last vaccination animals were challenged with GPCMV (TAMYC strain, 1 × 10^5^ pfu, SQ). A control group (*n* = 12) of seronegative (unvaccinated, green) animals were similarly challenged with virus. At 4, 8, 12 and 27 dpi, 3 animals per group were evaluated for viral load in target organs. Target organs: lung (**A**); liver (**B**); spleen (**C**) plotted as genome copies/mg tissue over 4, 8, 12 and 27 dpi. Salivary gland (**D**) was only evaluated at 27 dpi and plotted as genome copies/mg tissue. (**E**) Viremia at 4, 8, 12 and 27 dpi was plotted as genome copies/mL blood. Statistical analysis determined by unpaired Student’s *t* test; * *p* < 0.05; ** *p* < 0.005; ns = non-significant; # = DISCII vaccinated group value below the level of detection.

DISCII vaccinated animals from the previous section were subsequently challenged with wild type (strain TAMYC) GPCMV (1 × 10^5^ pfu, SQ), and a matching control group of unvaccinated animals (pre-screened GPCMV negative) were similarly challenged with TAMYC strain GPCMV. At subsequent time points (4, 8, 12 and 27 days post-infection, dpi), three animals per group were randomly selected for the evaluation of viral load (liver, lung, spleen and blood). The outcome (Figure 2) demonstrated that the DISCII vaccine did not provide sterilizing immunity to the TAMYC strain challenge.

Consequently, the DISCII vaccine had a more limited impact on virus dissemination in the vaccinated group. GPCMV (TAMYC strain) disseminated to all target organs in the vaccine group, but the viral load was reduced compared to the unvaccinated groups at all time points (Figure 2). The reduction in virus load was most effective in the spleen with the virus detected only at 12 dpi in the vaccine group, but present at all time points in the control group. The virus was detected in the blood at a constant level (approximately 10^3^ genome copies/mL blood) at 4, 8 and 12 dpi in the vaccine group, but peaked at earlier time points in the unvaccinated group at 4 and 8 dpi and was slightly above the vaccine group load at 12 dpi (Figure 2E). The virus continued to be detected in the salivary gland tissue at 27 dpi in vaccinated animals with a reduction of 2 logs compared to the unvaccinated group (Figure 2D). Results indicated a limitation of the current DISC vaccine strategy against cross strain heterologous virus protection compared to previous homologous protection studies against the 22122 strain challenge [38].

It was concluded that the DISCII vaccine strategy provided a limited ability to cross protect against the new strain virus challenge, and potentially would fail to completely prevent congenital infection, especially since there was a sustained viral load in the blood which would potentially enable significant placental infection in pregnant animals. 

### 3.3. Limitation of Cross Strain Protection in Animals Convalescent for 22122 Strain

**Figure 3:**
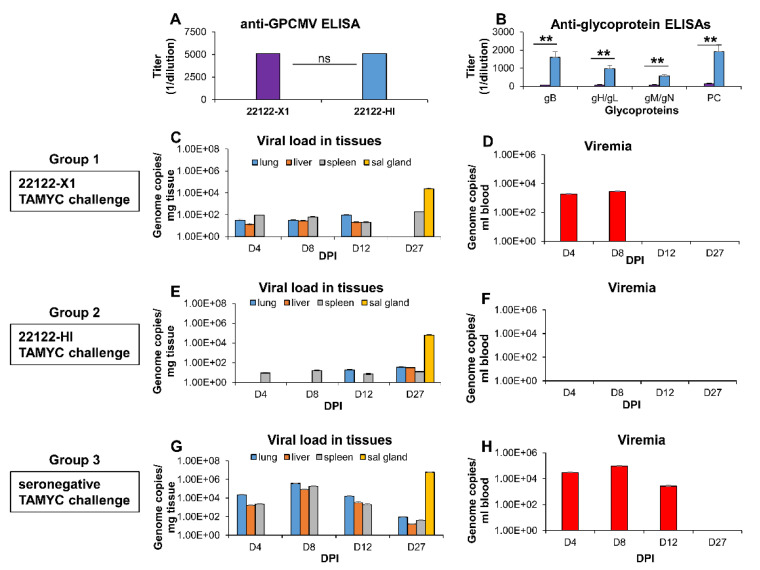
Comparative heterologous GPCMV (TAMYC strain) dissemination in convalescent (22122 strain) or control seronegative animals. Animals were infected with GPCMV (22122 strain) by single injection to establish convalescent natural immunity (22122-X1) or 3 sequential injections to establish hyperimmune status (22122-HI) prior to challenge (1 × 10^5^ pfu, SQ) with GPCMV (TAMYC strain). Convalescent animals were evaluated for anti-GPCMV ELISA titer and specific anti-glycoprotein ELISA titers. (**A**) Mean anti-GPCMV ELISA titer comparison of sera from animals in 22122-X1 group (purple) or 22122-HI group (blue). (**B**) Comparative mean anti-glycoprotein complex ELISA titers (gB, gH/gL, gM/gN, PC) from sera of animals in 22122-X1 (purple) or 22122-HI group (blue). Statistical analysis determined by unpaired Student’s *t* test; ** *p* < 0.005; ns = non-significant. (**C**–**H**) Comparative GPCMV (TAMYC strain) dissemination in convalescent animals: (**C**,**D**) group 1 (22122-X1); (**E**,**F**) group 2 hyperimmune (22122-HI); or (**G**,**H**) group 3 control seronegative animals. Animals (*n* = 12/group) were injected with 1 × 10^5^ pfu, SQ of GPCMV (TAMYC strain). On days 4, 8, 12 and 27 post infection (dpi), 3 animals from each group were evaluated for viral load in target organs (lung, liver and spleen), by real-time PCR of DNA extracted from tissue. Viral load plotted as viral genome copies/mg tissue. Salivary gland (sal gland) tissue was only evaluated at 27 dpi (**C**,**E**,**G**). Viremia detected at 4, 8, 12 and 27 dpi was plotted as genome copies/mL blood (**D**,**F**,**H**).

Next, we investigated if the DISCII vaccine provided higher level of protection compared to natural convalescent immunity against the 22122 strain, or animals hyperimmune to the 22122 strain. Animals that have been naturally infected with GPCMV or received a single dose of GPCMV to mimic natural infection induced a lower antibody response to viral glycoprotein complexes compared to animals given multiple injections of PC^+^ wild type virus to render them hyperimmune [12,38]. GPCMV seronegative animals (*n* = 12/group) were initially infected with either a single dose, to mimic natural infection, or animals received three sequential doses of the 22122 strain (1 × 10^5^ pfu, SQ) to render animals hyperimmune to GPCMV. Animals were evaluated for immune response at 2 months post-viral infection, and antibody titers are compared in Figure 3 between groups (22122-X1 and 22122-HI): Anti-GPCMV ELISA (Figure 3A); Anti-glycoprotein complexes (gB, gH/gL, gM/gN and PC) ELISAs (Figure 3B). Although anti-GPCMV titers were similar between groups (Figure 3A), the hyperimmune group had significantly higher antibody ELISA titers to all viral glycoprotein complexes (Figure 3B).

Subsequently, cross strain protection was evaluated in convalescent groups of animals (22122-X1 or 22122-HI) by challenging animals with TAMYC strain virus (1 × 10^5^ pfu, SQ). At subsequent time points post-infection (4, 8, 12 and 27 days post infection, dpi), three animals per group were randomly selected for evaluation of viral load (liver, lung, spleen and blood). The results demonstrated that the 22122-X1 group of animals (Figure 3C,D) had reduced the viral load in target organs compared to a control group of seronegative animals (Figure 3G,H). In the 22122-X1 group of animals, no virus was present in the blood by 12 dpi, and the virus was only detectable in the spleen and salivary gland by 27 dpi. However, the viral load in the salivary gland was still relatively high compared to control seronegative animals but with a 2 log reduction (Figure 3C,G). In hyperimmune animals (22122-HI), the viral load was substantially more reduced or non-detectable at different time points in target tissues (Figure 3E) and was below detection limits in the blood (Figure 3F). However, the virus continued to be shed in all tissues at 27 dpi with the viral load in salivary gland very similar to the viral load observed in the 22122-X1 convalescent group of animals. Overall, results indicated that the 22122 hyperimmune animals were better protected against the heterologous TAMYC virus challenge compared to the 22122-X1 convalescent animals or the DISCII vaccinated animals (Figure 2). However, the results indicate a limitation of cross strain protection by convalescent immunity from 22122 against re-infection by strain TAMYC, with the ability to prevent detectable viremia, but not virus dissemination to target organs.

### 3.4. Comparative Antibody Neutralization of 22122 and TAMYC GPCMV Strains by Hyperimmune Convalescent Sera from Animals (22122 or TAMYC Strain)

**Figure 4:**
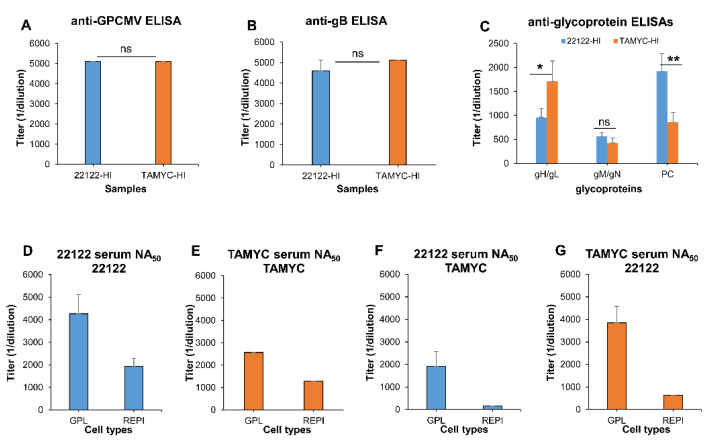
GPCMV hyperimmune convalescent sera antibody responses and virus neutralization on fibroblast and epithelial cells. (**A**–**C**) Comparative ELISAs of 22122-HI (blue) or TAMYC-HI (orange) sera. (**A**) Mean anti-GPCMV ELISA titers; (**B**) mean anti-gB glycoprotein ELISA titers; (**C**) mean anti-glycoprotein complex ELISA titers (gH/gL, gM/gN, PC) of sera from animals infected with GPCMV 22122 strain (22122-HI, blue) or TAMYC strain (TAMYC-HI, orange). (**D**–**G**) Comparative GPCMV neutralization (NA_50_) on GPL (fibroblast) or REPI (epithelial) cells by hyperimmune sera. (**D**) 22122-HI sera (blue) neutralization (NA_50_) of 22122 strain virus. (**E**) TAMYC-HI sera (orange) NA_50_ of TAMYC strain virus. (**F**) 22122-HI sera (blue) neutralization (NA_50_) of TAMYC strain virus. (**G**) TAMYC-HI sera (orange) NA_50_ of 22122 strain virus. Mean ELISA and neutralization values are a result of assay triplicates with each sample run a minimum of three independent times. Statistical analysis determined by unpaired Student’s *t* test; * *p* < 0.05; ** *p* < 0.005; ns = non-significant.

Since the 22122 strain-based immune response had a more limited impact against the heterologous TAMYC strain, we compared the antibody ELISA and neutralizing titers from the 22122 hyperimmune animals with the TAMYC hyperimmune animals. Specifically, we evaluated the ability of sera from hyperimmune animals (22122 or TAMYC) to neutralize homologous and heterologous virus infection on fibroblast and epithelial cells in an effort to determine if there was the potential for improvement of cross strain protection by a DISC vaccine strategy based on neutralizing antibodies. Pooled hyperimmune sera were derived from this current study (TAMYC strain hyperimmune sera) or from historical pooled sera (22122 strain hyperimmune sera) [38]. Figure 4 compares the pooled sera antibody ELISA titers from convalescent hyperimmune (22122 or TAMYC strain) animals: anti-GPCMV (Figure 4A), and specific glycoprotein complexes gB, gH/gL, gM/gM, and PC (Figure 4B,C).

ELISAs were based on the 22122 strain GPCMV coating antigen and specific glycoprotein complexes. Results demonstrate that regardless of the strain, the anti-GPCMV ELISA titer was similar between groups (5120), as were anti-gB (approximately 5000). However, there was more of a contrast with the anti-gH/gL titer, which was higher for the TAMYC strain (1707 vs. 960). The anti-PC was two-fold higher for 22122 compared to TAMYC (1920 vs. 853). The response to gM/gN was relatively low for both TAMYC and 22122 sera.

The ability of antibodies in convalescent hyperimmune (22122 or TAMYC strain) sera to neutralize (NA_50_) GPCMV (either 22122 or TAMYC strain) on fibroblast and epithelial cells were evaluated. Homologous neutralization of 22122 pooled sera against 22122 strain virus was most effective on GPL fibroblasts (titer = 4267) but had >two-fold lower NA_50_ titers on REPI epithelial cells (titer = 1920) (Figure 4D). The TAMYC sera of homologous TAMYC strain virus neutralization (Figure 1E) were also more effective on GPL fibroblasts (titer = 2560) compared to on epithelial cells (titer = 1280), with two-fold lower NA_50_ on REPI than on GPL cells. However, compared to 22122 sera homologous 22122 strain virus neutralization, titers on both fibroblast and epithelial cells were lower. In cross protective neutralization assays, the effectiveness of 22122 sera neutralization against heterologous TAMYC strain virus was evaluated (Figure 4F). Cross neutralization on both fibroblast (titer = 1920) and epithelial cells (titer = 160) was lower (Figure 4F) compared to homologous neutralization assays (Figure 4D). On GPL cells, the NA_50_ titer was >two-fold lower, while the titer on REPI cells decreased by 12-fold. The reverse comparative evaluation of TAMYC sera of the heterologous 22122 strain was evaluated (Figure 4G). TAMYC sera were more effective against the 22122 strain on GPL (titer = 3840) compared to REPI cells (titer = 640), Figure 4G. TAMYC sera had three-fold lower NA_50_ titers against the 22122 strain on epithelial cells, compared to the 22122 sera of the 22122 strain (Figure 4D,G). Overall, it was concluded that convalescent hyperimmune sera had the ability to neutralize virus infection but worked best on fibroblast cells compared to epithelial cells. Additionally, virus neutralizations of the 22122 strain by homologous sera (22122) and TAMYC sera (heterologous) were highly effective especially on fibroblast cells where NA_50_ titers were similar. In contrast, homologous and heterologous sera were less effective against the TAMYC virus, with 22122 sera particularly limited in NA_50_ titers against TAMYC epithelial infection (Figure 4F), compared to homologous NA_50_ titers (Figure 4G) against TAMYC on REPI cells. This potentially indicated that a DISC vaccine strategy based on strain 22122 is likely to be less effective against the GPCMV TAMYC strain virus challenge or similar clinical strain which is supported by vaccine protection outcome findings (Figure 2). Overall, a DISC vaccine based on a clinical strain (TAMYC) might have better efficacy against GPCMV homologous and heterologous infections. Potentially, this indicates a limitation of the current HCMV DISC vaccine strategy, which is based on a lab-adapted AD169 HCMV strain [47].

## 4. Discussion

An important requirement of an effective CMV vaccine is an ability to provide protection greater than that of convalescent natural immunity because the risk of congenital CMV is not only by primary infection during pregnancy but also from re-infection by new strains of the virus. Indeed, it is the ability to attain significant cross strain protection that likely increases the challenge for a successful vaccine against congenital CMV. Although preclinical animal models are available for the evaluation of CMV vaccine efficacy, they potentially suffer from various factors that reduce the translational impact. Firstly, HCMV cannot be directly studied in an animal model because of species specificity, which precludes direct evaluation of HCMV that might be available for other viral pathogens in animals models (e.g., influenza virus) [55]. Consequently, animal species-specific CMV studies are necessary. Although mouse models of murine cytomegalovirus have the advantage of powerful immunological tools, and gene knockout animals, there is a lack of strain variation in MCMV at the amino acid protein coding sequence level which limits the ability to evaluate the impact of viral strain infection on vaccine protection. The most commonly studied strains (K181 and Smith) are identical at the protein level [56]. Furthermore, MCMV does not cause congenital CMV, nor does it encode a PC. MCMV encodes a second gH-based trimer more similar to Epstein-Barr virus [57], which affects the translational impact of MCMV vaccine studies.

Although a NHP congenital CMV model with RhCMV has recently been developed for congenital CMV [58], no vaccine protection studies against vertical transmission have been evaluated. Importantly, RhCMV encodes a homolog PC that is required for virus tropism to non-fibroblast cells and pathogenicity in the NHP model [59]. Interestingly, a hyperimmune globulin (HIG) therapy strategy in the NHP applied to pregnant CD4+ T cell-depleted animals resulted in protection against congenital RhCMV, which implied that neutralizing antibodies were sufficient to protect against congenital CMV, but protection required inclusion of potent antibodies to PC [60]. In contrast, HIG therapy against congenital CMV in a human clinical trial did not reach statistical significance [61,62]. Potentially, protection against congenital RhCMV may have requirements different from those for protection against congenital HCMV. However, in the congenital RhCMV studies, the virus was introduced by intravenous route, rendering it cell free and potentially easier to rapidly neutralize, which likely weakens the study. The major focus of RhCMV vaccine research with the NHP model has been on horizontal transmission with varying levels of success, but no specific approach has attained sterilizing immunity [63]. Although a DISC vaccine strategy has been developed for RhCMV, it is based on the knockout of the gL glycoprotein gene which forms the basis of two important gH-based glycoprotein complexes (gH/gL/gO and PC) that impact the immune response to these entry complexes [64]. The availability of various RhCMV strains enables the potential for evaluation of cross strain protection to a level more similar to that of the HCMV infection of humans. However, a significant limitation to this NHP model is the small number of available CMV negative animals, as well as the prohibitive cost involved that precludes high throughput vaccine studies against congenital CMV.

Consequently, the guinea pig remains an important model for the development of intervention strategies against congenital CMV. This animal model suffers from limitations associated with available reagents which impact various human disease models based on this animal. However, the recent in-depth sequencing of the guinea pig genome has enabled the application of the CRISPR/Cas9 gene knockout strategy, as well as the ability to generate synthetic genes for cellular innate immune studies [38,39,46,65]. Furthermore, our laboratory and other investigators have established novel non-fibroblast guinea pig cell lines for virus tropism studies. However, an additional limitation of this model has been the use of a single strain of GPCMV (22122), which was isolated more than 50 years ago and passaged multiple times on fibroblasts, increasing the likelihood of adaptation that potentially limits the pathogenicity of this virus, despite the ability to cause congenital infection. The lack of additional GPCMV strains available prevented an ability to evaluate cross strain protection by candidate CMV vaccine strategies. We consider the latter an important benchmark for advancement of any CMV candidate vaccine, and this was compounded by the recent milestone achievement of complete protection against congenital GPCMV (22122 strain) by the use of two different candidate vaccine approaches: DISC vaccine [38]; and interferon sensitive attenuated live vaccine strain [66]. The recent isolation of a new strain of GPCMV (TAMYC) [12] without passage on fibroblast cells has enabled the bar to be raised for vaccine efficacy studies in this model by the evaluation of cross strain protection by utilizing the TAMYC strain virus. As with clinical HCMV strains, the greatest range of sequence variation between 22122 and TAMYC strains is the gO protein with 25% difference at the predicted amino acid level [12]. Other viral glycoproteins additionally differ between strains but the difference is not as profound and similar to that seen between HCMV strains [12]. Importantly, the TAMYC strain virus encodes a PC similar to the 22122 strain virus [12].

The correlates of protection against congenital CMV are poorly understood, but it is thought that antibody response is a significant driver for protection. The gB protein is essential for both HCMV and GPCMV infection of all cell types and an immunodominant antibody target. Consequently, gB has been a central focus or corner stone of many vaccine strategies against CMV both in preclinical and clinical studies. However, a standalone gB vaccine fails to achieve better than 50% efficacy in the guinea pig or in clinical trials. A limitation of gB vaccine efficacy in the guinea pig model was, until recently, the use of various truncated gB constructs. These strategies lacked the ability to form a trimeric complex found on the virion, and therefore limited the vaccine neutralizing titer because of the absence of higher order antigens. We recently evaluated recombinant Ad vector vaccines encoding either GPCMV gB lacking a transmembrane domain or a full length gB. Although both vaccines produced similar high antibody titers, the full length gB vaccine (AdgBWT), capable of forming a trimeric complex, produced higher neutralizing titers on both fibroblast and non-fibroblast cell types [27]. The current DISC vaccine is capable of both monomeric and trimeric gB complex formation; the antibody titer is lower than that of the AdgB vaccine, but is considered to have similar anti-gB neutralizing titers to that of hyperimmune convalescent animals based on anti-gB sera absorption studies [38]. In HCMV, non-neutralizing gB antibodies also contribute to gB vaccine efficacy, and it is likely that the DISC vaccine also produces non-neutralizing antibodies not only to gB but to other viral antigens. The impact of non-neutralizing gB antibodies for GPCMV would appear to have a minimal impact on vaccine protection [27], but remains to be more fully evaluated in future studies. However, it is clear that the gB immune response is limited in efficacy as a vaccine candidate failing to fully protect against GPCMV (22122 strain), despite improvements in neutralizing titers. Additionally, the limitation of a gB-only based vaccine strategy is further compounded when evaluated for cross strain protection against the TAMYC strain virus, despite 99% similarity in amino acid sequence [48]. Importantly, the TAMYC strain virus is more similar to clinical HCMV strains, highly cell associated and preferentially is tropic for non-fibroblast cells, in contrast to 22122 strain GPCMV [12]. The failure of the gB vaccine to cross protect between strains has also been demonstrated in the RhCMV NHP horizontal transmission model [67], which further indicates the limitation of gB as a standalone CMV vaccine candidate.

In HCMV, the PC is a potent neutralizing target antigen in convalescent-phase patients and in vaccine studies [40,41,68]. In GPCMV, the PC is necessary for virus tropism to non-fibroblast cells (including epithelial, endothelial and trophoblasts) and congenital infection. Furthermore, inclusion of the PC in a GPCMV DISC vaccine strategy improved virus neutralization on non-fibroblast cells by generation of PC-specific neutralizing antibodies [38]. This resulted in complete protection against congenital CMV (22122 strain) in this animal model, as well as sterilizing immunity compared to a previous DISC vaccine lacking PC [24,38]. Additionally, the inclusion of the PC in a live attenuated GPCMV vaccine strategy also resulted in complete protection against congenital CMV [66]. These recent vaccine studies demonstrated the importance of the PC to improve vaccine efficacy in this animal model. Both approaches induce an immune response to all glycoprotein complexes, but the inclusion of the PC in the vaccine design dramatically improved the protective immune response [24,38,66,69]. However, the DISCII vaccine sera did not reach the level of virus neutralization on both fibroblast and epithelial cells against the 22122 strain observed for hyperimmune sera from animals convalescent for GPCMV (22122 strain). This suggests room for improvement of the vaccine neutralizing titer, more especially since the DISCII vaccine was less effective at neutralizing the heterologous TAMYC strain virus on both fibroblast and epithelial cells compared to the 22122 strain NA_50_ titers. Likely, there is a limitation of virus neutralization evoked by cross strain protection from the 22122 strain background, since the 22122 strain hyperimmune sera are less effective against the TAMYC virus. In contrast, TAMYC hyperimmune sera are more effective against the TAMYC virus, especially on epithelial cells with higher neutralizing titers, than against the 22122 strain. Potentially, this indicates that a DISC vaccine built in the backdrop of the clinical TAMYC strain may have better efficacy and cross strain protection, but awaits future study since no recombinant virus has been generated based on the TAMYC strain. This might also indicate a potential failing of an HCMV DISC vaccine strategy based on the backdrop of the AD169 strain; a fibroblast adapted HCMV strain with restored ability to express the PC [70]. Strain-specific neutralizing target antigens have been identified in HCMV, and most recently in gH [71], which are likely to impact vaccine efficacy.

As with an RhCMV gB-based vaccine strategy, an RhCMV PC-based vaccine failed to provide complete protection against horizontal viral transmission [72]. Potentially, this indicates a general failing of a gB or a PC-based standalone vaccine strategy which does re-enforce the advantage of a DISC vaccine approach. Recently, a potent therapeutic antibody was identified that targets both gB and PC in HCMV, which indicates the likely interdependent importance of both of these antigens as neutralizing targets and the value of including both antigens in CMV vaccine design [73]. Potentially, CMV DISC vaccine efficacy could be enhanced by the inclusion of gB or gH, and unique PC ORFs from various important divergent viral strains to improve cross strain protection. This is a possible avenue for study in future HCMV or animal CMV DISC vaccine design. It is important to note that the TAMYC strain mainly infects by cell-cell spread with limited levels of cell release virus, similar to HCMV clinical strains. Potentially, cell-cell spread limits the effect of neutralizing antibodies and may serve as an effective escape mechanism from the host antibody response.

In convalescent CMV immunity, the T cell response is considered to be important and likely contributes to protection against congenital CMV. Indeed, the evasion of CD8 T cell response is critical for CMV superinfection [74]. In addition to antibody response, the GPCMV DISC vaccine strategy has previously been shown to evoke a cell-mediated response against pp65 tegument protein (GP83) in both PC^+^ and PC^−^ DISC vaccines [24,38]. In HCMV, pp65 is thought to be the immunodominant T cell target, but this may not equate with the most effective target antigen for a protective T cell response. The non-structural IE1 protein in HCMV induces a T cell response and has been demonstrated to be partially protective in RhCMV studies [75]. Potentially, other viral antigens also induce a cell-mediated immune response in GPCMV such as GPCMV IE1 [65], and this is currently under evaluation. In both HCMV and GPCMV, the pp65 tegument protein is an innate immune evasion factor targeting cGAS and IFI16 [46,76]. The GP83 cell-mediated response has been demonstrated to be partially protective in various vaccine strategies against GPCMV [30,45,46]. However, the protective effect of the GP83 antigen T cell response is further limited in the context of cross strain GPCMV protection. In a recent Ad vector vaccine study encoding GPCMV GP83 (AdGP83), we demonstrated AdGP83 induced a cell-mediated immune response similar to GPCMV convalescent and DISC vaccinated animals; however, cross strain protection against the TAMYC challenge virus in AdGP83 vaccinated animals was relatively poor compared to protection in the 22122 challenged animals [46]. This was despite a 100% identity in the predicted GP83 amino acid sequence between the GPCMV 22122 and TAMYC strains. Thus, although the DISC vaccine has been demonstrated to generate a cell mediated response to GP83, it is unlikely to be an effective cross protective antigen based on the AdGP83 based vaccine studies [46]. An ability to comprehensively evaluate the T cell response against GPCMV in the guinea pig is currently lacking, and is a limitation of studies in this model, and should be a focus for future development.

## 5. Conclusions

In conclusion, the current GPCMV DISC vaccine strategy, although highly successful against the homologous strain (22122 strain) virus challenge dissemination and congenital infection in the animal, fails to provide high level protection against the heterologous virus (TAMYC strain) challenge. Consequently, the current version of the DISC vaccine would be unlikely to provide a high level of protection against congenital infection, more especially given the sustained viral load in the blood in the TAMYC virus challenged vaccinated animals. The ability for a CMV vaccine to cross protect against infection by a new strain of virus is an important additional stage of evaluation for any pre-clinical CMV vaccine. Studies in GPCMV hyperimmune immune convalescent animals suggest that the efficacy of the current DISC vaccine can be improved, but this is likely to require modifications to the DISC vaccine to enhance both the antibody and cell-mediated immune response. Since the DISC vaccine virus is cloned as an infectious BAC plasmid, additional modifications are easily attained via modifications of the viral genome in bacteria. Overall, the current results suggest that an HCMV DISC vaccine strategy will also likely require additional modifications to maximize cross strain protection, which becomes a significant factor in areas or groups endemic for HCMV and the potential exposure to multiple strains of the virus.

## Data Availability

Not applicable.
